# The Roles of Individual Mammalian Argonautes in RNA Interference *In Vivo*

**DOI:** 10.1371/journal.pone.0101749

**Published:** 2014-07-03

**Authors:** Vera M. Ruda, Rohit Chandwani, Alfica Sehgal, Roman L. Bogorad, Akin Akinc, Klaus Charisse, Alexander Tarakhovsky, Tatiana I. Novobrantseva, Victor Koteliansky

**Affiliations:** 1 Koch Institute for Integrative Cancer Research, Massachusetts Institute of Technology, Cambridge, Massachusetts, United States of America; 2 Alnylam Pharmaceuticals, Cambridge, Massachusetts, United States of America; 3 Laboratory of Immune Cell Epigenetics and Signaling, Rockefeller University, New York, New York, United States of America; Universidade Federal do ABC, Brazil

## Abstract

Argonaute 2 (Ago2) is the only mammalian Ago protein capable of mRNA cleavage. It has been reported that the activity of the short interfering RNA targeting coding sequence (CDS), but not 3′ untranslated region (3′UTR) of an mRNA, is solely dependent on Ago2 *in vitro.* These studies utilized extremely high doses of siRNAs and overexpressed Ago proteins, as well as were directed at various highly expressed reporter transgenes. Here we report the effect of Ago2 *in vivo* on targeted knockdown of several endogenous genes by siRNAs, targeting both CDS and 3′UTR. We show that siRNAs targeting CDS lose their activity in the absence of Ago2, whereas both Ago1 and Ago3 proteins contribute to residual 3′UTR-targeted siRNA-mediated knockdown observed in the absence of Ago2 in mouse liver. Our results provide mechanistic insight into two components mediating RNAi under physiological conditions: mRNA cleavage dependent and independent. In addition our results contribute a novel consideration for designing most efficacious siRNA molecules with the preference given to 3′UTR targeting as to harness the activity of several Ago proteins.

## Introduction

RNAi and in particular siRNA technology has advanced from bench to bedside in under a decade [1], [2]. siRNA utilizes a natural pathway that cells designed for miRNA induced gene modulation and antiviral protection. While interactions between miRNAs and the proteins which constitute RNA-induced silencing complex (RISC) have been well studied [3], [4], including RISC loading [5]–[7] and resulting complex structure [8]–[10], the interactions of exogenous siRNAs with Argonaute proteins are less well understood. Functional interaction of siRNA with Ago2 has been demonstrated by the loss of knockdown in the absence of Ago2 or in the presence of a Slicer-incompetent Ago2, and by the reconstitution of the Slicer activity with purified wild-type Ago2 protein [11], [12]. Little is known about functionality of siRNA binding to the other three mammalian Argonaute family members. Some residual knockdown in the absence of Ago2 has been reported by several groups [6], [13], [14], while others have not seen response to siRNA treatment in cells lacking Ago2 [11]. All four Ago proteins encoded in the mammalian genome are expressed in most tissues and cultured mammalian cell lines, although in different proportions [15]. They have all been shown to bind miRNAs and siRNAs indiscriminately of sequence [11], [12], [16], [17], to interact with a common set of helicases and mRNA-binding proteins, including the three TNRC6 proteins (−A,−B,−C) [18], [19] and to localize to P-bodies in mammalian cells, with a capability of targeting mRNAs to the general eukaryotic machinery for translation control and mRNA degradation [20]–[22]. This is true for Ago3 and Ago4 as well, though they do not possess either passenger strand cleavage activity or siRNA strand dissociating activity [5].

Thus, the question remains: does a siRNA assembled in RISC with a non-Slicer Argonaute protein within a mammalian cell pair with mRNAs to contribute to post-transcriptional repression by translation inhibition and/or targeting the mRNA for degradation, similar to miRNA [20], [23]–[27]? Do the functions of Ago1, Ago3, and Ago4 differ from those of Ago2 [27], [28]? Or do these complexes compete with Ago2-containing RISC for the target mRNA sites and/or siRNA reducing potency of the siRNA [13], [29]? It is likely that both alternatives are true under different conditions: (i) Excess of non-Slicer Argonautes may effectively compete with Ago2, decreasing target mRNA knockdown, as has been shown for shRNA [13]; (ii) in the absence of Ago2 non-Slicer Argonautes may provide sufficient miRNA-guided knockdown to sustain cell viability [30], if not full ontogenesis (as germline mutation of the *Ago2* gene is embryonic lethal [11], [31]); and (iii) in some cases non-Slicer Argonautes may cause target-specific siRNA mediated mRNA degradation [13], [14], [27].

Until now, the limited number of genes tested and insufficient transcript coverage by targeting siRNAs have impeded the understanding of the role of siRNA’s sequence and target site position within the transcript, which led to controversial results and hypotheses. Several groups have shown previously that some of siRNAs retain part of their on-target activity in the absence of Ago2 [6], [13], [14]. These data came from experiments with transgenic targets and only one siRNA per target, leaving an open question whether this effect is due to the nature of the target or some features of siRNAs, such as sequence or position of the target site within the mRNA.

We are the first to have systematically analyzed the effect of Ago2 absence on knockdown of endogenous genes by siRNAs, targeting coding sequence (CDS) and 3′ untranslated region (3′UTR) of several genes, including knockdown with siRNA pairs targeting CDS and 3′UTR of three genes: coagulation factor VII (*FVII*), fatty acid desaturase 1 (*Fads1*), and Ras-related protein Rab-5C (*Rab5c*) tested *in vivo*. We demonstrate complete loss of activity of the siRNAs targeting CDS regions of these three genes in the absence of Ago2 in mouse liver. We show persistence of knockdown by siRNAs targeting 3′UTRs of the same three genes, and that both Ago1 and Ago3 proteins present in physiological amounts contribute to residual knockdown observed in the absence of Ago2 in liver. Ago1 and Ago3 (and possibly Ago4 in tissues, where it is more abundant) are involved in 3′UTR-targeted siRNA-mediated knockdown of mRNA (indicating potential benefits of targeting 3′UTR versus CDS).

## Results and Discussion

### siRNAs targeting CDS and 3′UTR differ in their Ago2 dependence *in vitro*

**Figure 1:**
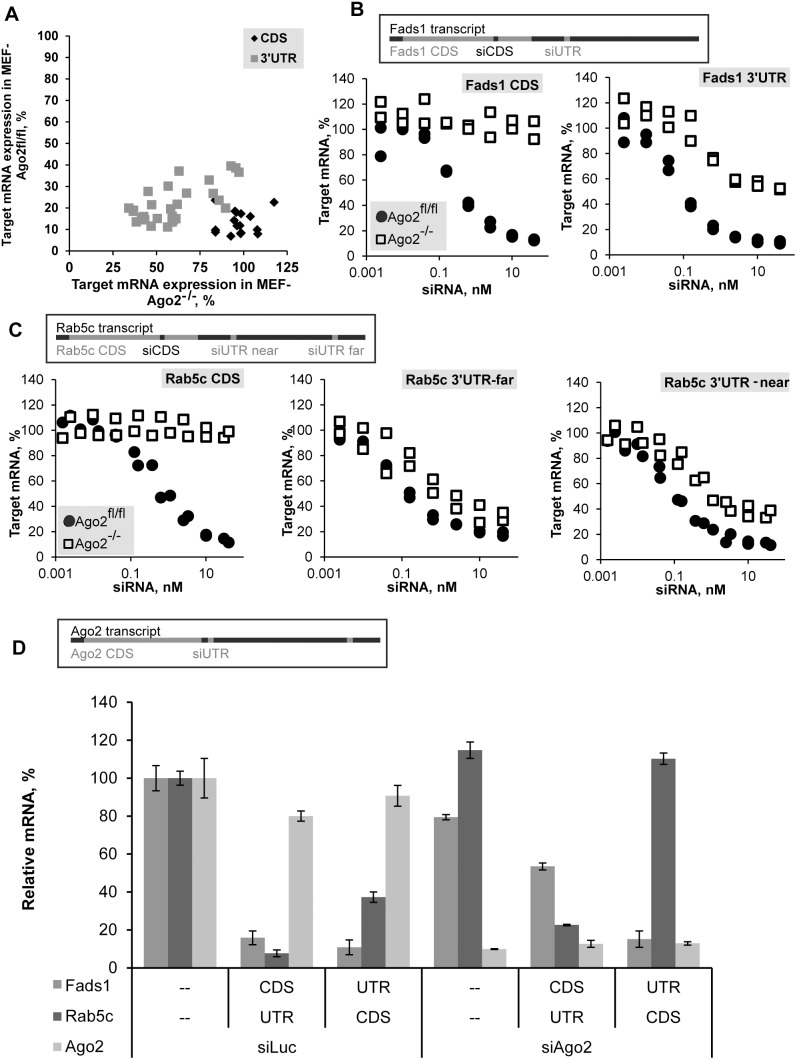
Differential Ago2 dependence of siRNAs targeting coding sequence (CDS) and 3′-untranslated region (3′UTR) of mRNA *in vitro*. (A) Mouse embryonic fibroblast (MEF) cells, Ago2^fl/fl^ and Ago2^−/−^ (wild-type and Ago2 knockout) were transfected with 10 nM of siRNAs targeting CDS or 3′UTR (16 or 28 siRNAs, respectively, Table S1) of mRNAs of four genes: Fads1, Fads2, Rab5a, and Rab5c. Levels of expression of target-gene mRNA in Ago2^fl/fl^ MEF cells and Ago2^−/−^ MEF cells measured by branched DNA (bDNA) assay 24 h post-transfection were plotted as percentage of relative target mRNA compared to Luciferase siRNA transfected controls. (B, C) Ago2^−/−^ MEF cells (square) or Ago2^fl/fl^ MEF cells (circle) were transfected with siRNAs targeting CDS or 3′UTR of Fads1 (B) or Rab5c (C) mRNA (relative positions of target sites within mRNA are shown). Transfection and assay as described in A, but dilution series with the maximum dose of 40 nM were done. Combined results of two independent transfections are shown. (D) Ago2^fl/fl^ MEF cells were transfected with 10 nM siRNA targeting 3′UTR of Ago2 (Eif2c2) mRNA or Luciferase, followed by transfection with two combinations of siRNAs targeting Fads1 and Rab5c (CDS-targeting for one gene and 3′UTR-targeting for the other gene, 10 nM each, or 20 nM of Luciferase siRNA control) 48 hours later. Levels of target-genes and Ago2 mRNA expression measured by bDNA assay 24 h after second transfection were plotted as percentage of relative target mRNA compared to Luciferase siRNA-transfected control (mean ± s.d., n = 3). Legend in the bottom left corner of the graph also indicates the line in the X-axis describing the treatment type (none, CDS, 3′UTR) by target gene.

In order to systematically analyze the role of Ago2 in the knockdown mediated by exogenous siRNA we tested the activity of a collection of siRNAs targeting different regions of four endogenous gene transcripts (*Fads1, Fads2, Rab5a,* and *Rab5c*; sequences of duplexes are listed in Table S1) in mouse embryonic fibroblast (MEF) cells expressing the wild-type Ago2 (MEF harboring Ago2 flanked by LoxP sites, Ago2^fl/fl^) or its truncated inactive variant (Ago2^−/−^) [31]. Indeed, significant part of siRNAs retained silencing activity in the absence of Ago2. Mapping of their target site positions showed that only siRNAs targeting 3′UTR remained active, while all tested siRNAs targeting CDS did not cause any knockdown in the absence of Ago2 (Fig. 1A). All 16 duplexes targeting CDS showed robust knockdown (>75%) in Ago2^fl/fl^ MEF cells, while showing no significant knockdown (<20%) in Ago2^−/−^ MEF cells. At the same time, 21 of 28 duplexes targeting 3′UTR retained activity in Ago2^−/−^ cells, this effect was observed even for the less potent siRNAs (which caused only ∼60%–70% target knockdown in control cells). This effect appears to be independent of the strength and sequence of siRNA, as several different duplexes with IC50 concentration ranging from 0.05 nM to 0.8 nM, targeting the 3′UTR of the same gene, retain partial activity. Furthermore, since the same was seen for four targeted genes, it is evidently not a gene-specific effect either. It was recently shown that miRNA activity is inhibited in translated regions of transcripts and thus miRNAs preferentially target 3′UTR [32], our data suggest that in the absence of Ago2 exogenous siRNAs follow the same rules.

Next we sought to determine the maximum level of knockdown that can be achieved in the absence of Ago2 and to verify absence of knockdown with duplexes targeting CDS at higher concentrations of siRNA. To this end we transfected Ago2^−/−^ or Ago2^fl/fl^ MEF cells with increasing concentrations of siRNAs targeting CDS or 3′UTR of two genes: *Fads1* and *Rab5c*, selected from the set used in the first experiment, based on their high activity in the presence of Ago2 (these duplexes are bold in Table S1). All tested siRNAs reached saturation at 40 nM or lower in Ago2^fl/fl^ MEF cells (Figures 1B and C). The duplexes targeting CDS did not demonstrate any activity in Ago2^−/−^ MEF cells even at this maximal dose. All of the tested duplexes targeting 3′UTR showed dose-dependent knockdown, irrespective of their relative positions within 3′UTR, reaching saturation at the highest dose tested both in control and Ago2^−/−^ MEF cells. At the same time, the depth of achievable knockdown in Ago2^−/−^ MEF cells was always less profound, than in control cells, in agreement with what was shown previously [14]. Just as duplexes differ in their effectiveness in the presence of Ago2, the depth of knockdown observed in Ago2^−/−^ MEF cells also varied. It may be dependent on the efficiency of loading and activation of particular siRNAs in RISC containing different non-Slicer Argonaute proteins [6].

### Role of Ago2 in CDS- and 3′UTR-targeted knockdown

We further validated the results obtained in Ago2^−/−^ MEFs by transiently reducing levels of Ago2 with siRNA against Ago2. We developed siRNA targeting the 3′UTR of Ago2 transcript allowing sustained Ago2 knockdown in wild-type cells. To confirm the role of Ago2 in knockdown directed by siRNAs targeting CDS or 3′UTR, we utilized double-transfection strategy described by [14], [33]. We transfected Ago2^fl/fl^ MEF cells with siRNA targeting Ago2 mRNA, followed in 24 hours by transfection with a mixture of siRNAs targeting CDS and 3′UTR used earlier in the experiments with knockout cells (Fig. 1D). Since MEF cells have been shown to divide rapidly [34] and therefore dilute transfected siRNA fast, the transfection with *Fads1* and *Rab5c* siRNAs mixes was combined with second siLuc (control siRNA targeting Luciferase mRNA) or siAgo2 treatment, to ensure sustained downregulation of Ago2 protein. In this experiment siRNA targeting CDS of *Fads1* mRNA was combined with equal amount of siRNA targeting 3′UTR of *Rab5c* mRNA, or siRNA targeting 3′UTR of *Fads1* mRNA was combined with siRNA targeting CDS of *Rab5c* mRNA, to control effect of siRNAs targeting different regions of transcripts in the same cells simultaneously. Ago2 siRNA treatment led to over 80% reduction in Ago2 mRNA and a decrease in efficacies of all *Fads1* and *Rab5c* siRNAs tested, with the more pronounced effect on duplexes targeting CDS: the knockdown of *Fads1* mRNA was significantly decreased and in the case of *Rab5c* mRNA the knockdown was lost. Thus, the phenotype observed in Ago2^−/−^ MEF cells, namely the loss of CDS-targeted knockdown and weakening of activity of siRNA targeting 3′UTR may be attributed to the absence of Ago2. Our results are in agreement with previous data where levels of Ago2 were downregulated using a treatment with RNase H-dependent antisense oligonucleotides [14].

To test whether catalytic activity of Ago2 is indispensable for the knockdown mediated by siRNA targeting CDS we introduced wild-type Ago2 or Ago2 (D669A), which is catalytically inactive Slicer-incompetent mutant [35] in Ago2^−/−^ MEF cells, followed by transfection with the same siRNA duplexes targeting CDS or 3′UTR of *Fads1* mRNA as used above. Only the expression of the wild-type Ago2, but not the catalytically inactive mutant Ago2 restored CDS-targeted knockdown (Figure S1). Possible difference in mechanisms of action of miRNA targeting CDS and 3′UTR was predicted based on patterns of their conservation, suggesting unique role of Ago2 in CDS-targeted knockdown by miRNA [36]. These data combined with our observation of complete dependence of siRNA targeting CDS on Ago2 led us to hypothesize that Ago2 has a unique role in CDS-targeted knockdown by siRNA. Failure of the particular mutant Ago2 to rescue the Ago2^−/−^ phenotype may indicate but does not imply Slicer enzymatic activity of Ago2 as prerequisite for inhibition of targets with CDS sites. D669A substitution may modify overall functionality of Ago2 in addition to Slicer activity. This merits further investigation and can be tested by comparison of various Slicer-inactive mutants in similar rescue experiments.

### Cleavage-independent siRNA-directed mRNA knockdown in the absence of Ago2

**Figure 2:**
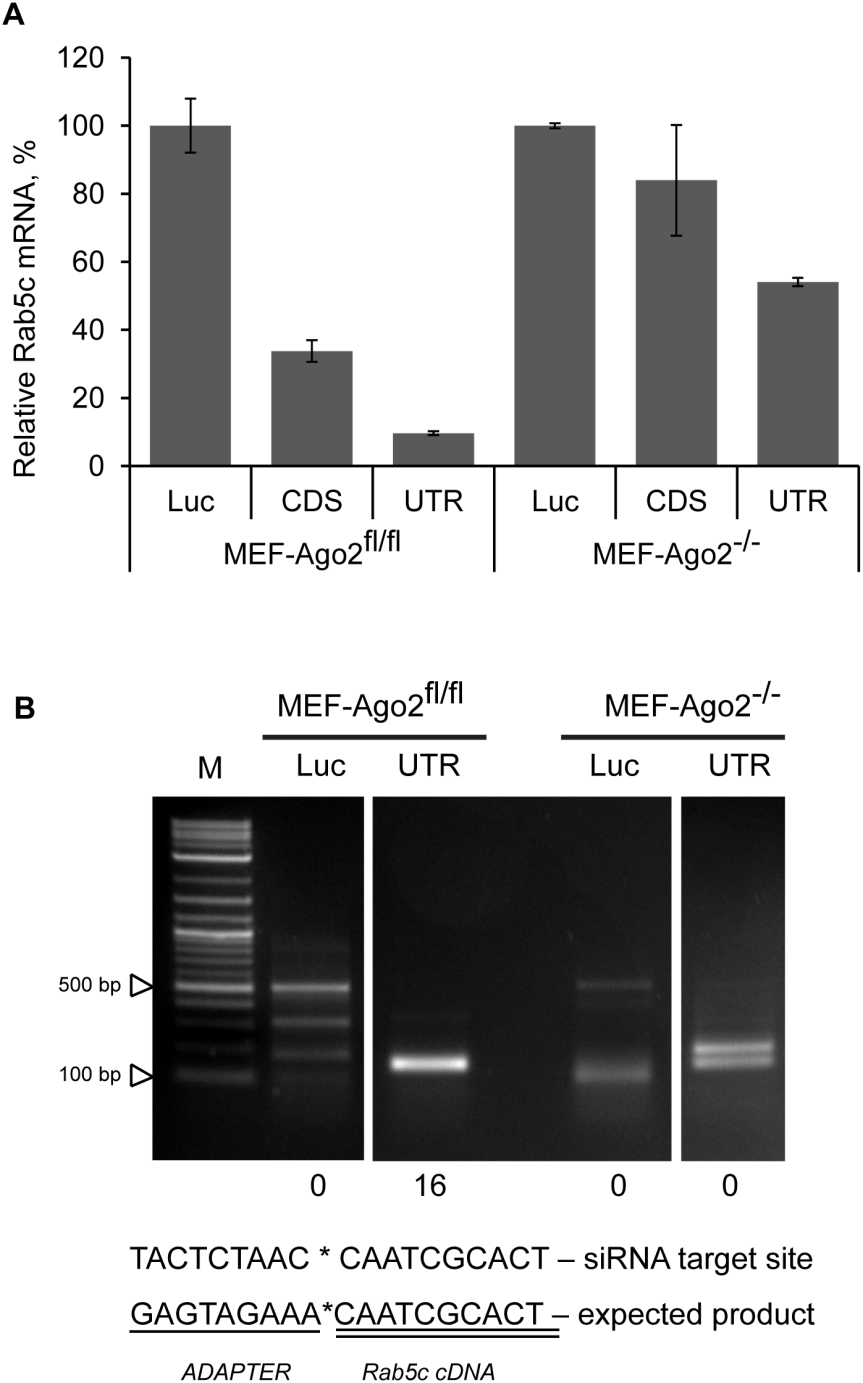
Cleavage independent siRNA-mediated degradation of mRNA in the absence of Ago2. Ago2^fl/fl^ MEF cells and Ago2^−/−^ MEF cells were transfected with 10 nM of siRNAs targeting Luciferase, or CDS, or 3′UTR of the Rab5c mRNA. bDNA assay (A) and 5′RACE (B) were performed on total RNA isolated from cells 3 h post-transfection. (A) Data is presented as mean ± s.d. for two technical replicates of bDNA measurement. (B) 5′RACE nested PCR detection of cleavage product. Numbers of clones bearing expected inserts are indicated below corresponding lanes of the gel (expected PCR product length is 145 bp; expected adapter/Rab5c junction sequence is shown below; 16 clones were sequenced for each PCR reaction).

To establish if reduction in a transcript level in the absence of Ago2 depended on Slicer, we performed 5′RACE on total RNA isolated from Ago2^fl/fl^ MEF cells and Ago2^−/−^ MEF cells transfected with specific (*Rab5c*-3′UTR-near) or control (Luciferase) siRNA for 3 hours. Prior to 5′RACE knockdown in the RNA samples was verified by branched DNA (bDNA) assay (Fig. 2A). siRNA targeting the 3′UTR of *Rab5c* caused considerable knockdown in both control and Ago2^−/−^ MEF cells even at such an early time point after transfection. The knockdown level reached by 24 hours post-transfection with siRNA targeting 3′UTR was a lot deeper in both types of cells, while in Ago2^−/−^ MEF cells transfected with siRNA targeting CDS there was no significant mRNA knockdown even at 24 hour time point (Figure S2A). Interestingly, knockdown seems to develop slower in the absence of Ago2, as seen by comparison of 3 h-long and 24 h-long treatments, supporting a miRNA-like mechanism, where translational inhibition precedes mRNA degradation [20].

PCR products of the 5′RACE were visualized on agarose gel (Fig. 2B and Fig. S2B), gel purified, cloned, and sequenced. All of clones sequenced from the Ago2^fl/fl^ MEF cells transfected with specific (*Rab5c*-3′UTR-near) siRNA mapped to the predicted cleavage site, while none of the clones from Ago2^−/−^ MEF cells did (Figure S3). Instead, in several independent amplification reactions we have detected products of variable lengths, similar to products found in some of the control Luciferase siRNA treated samples, which are characteristic of random mRNA breaks in the absence of Ago2 siRNA-specific targeting, and suggest target-site cleavage independent miRNA-like mechanism of mRNA degradation (Figure S3 and data not shown). The same mechanism has been shown for siRNA mediated Ago2- and Dicer-independent off-target degradation of mRNA [6], [14]. Interestingly, most of the 5′RACE products from Ago2^−/−^ MEF samples treated with *Rab5c*-3′UTR-near siRNA mapped to *Rab5c* mRNA, while very few of the 5′RACE products from samples treated with siRNA targeting Luciferase mRNA mapped to *Rab5c* mRNA. This indicates that there are more *Rab5c* mRNA degradation products, which can be ligated to the adapter and amplified (mRNA needs to be decapped, broken, or cleaved by an endonuclease to obtain a phosphate-free 5′end and become suitable substrate for T4 RNA ligase) in the presence of specific siRNA, even in the absence of Ago2 (Supplemental Figure S3 and data not shown). Thus, Ago2 is indispensable for siRNA-mediated cleavage of mRNA, while significant gene knockdown can be achieved by siRNA treatment in the absence of Ago2, by other means of RISC-dependent mRNA degradation [22], [25], [27].

### *In vivo* CDS-targeted knockdown is fully dependent, while 3′UTR-targeting siRNA is *partially dependent* on Ago2

**Figure 3:**
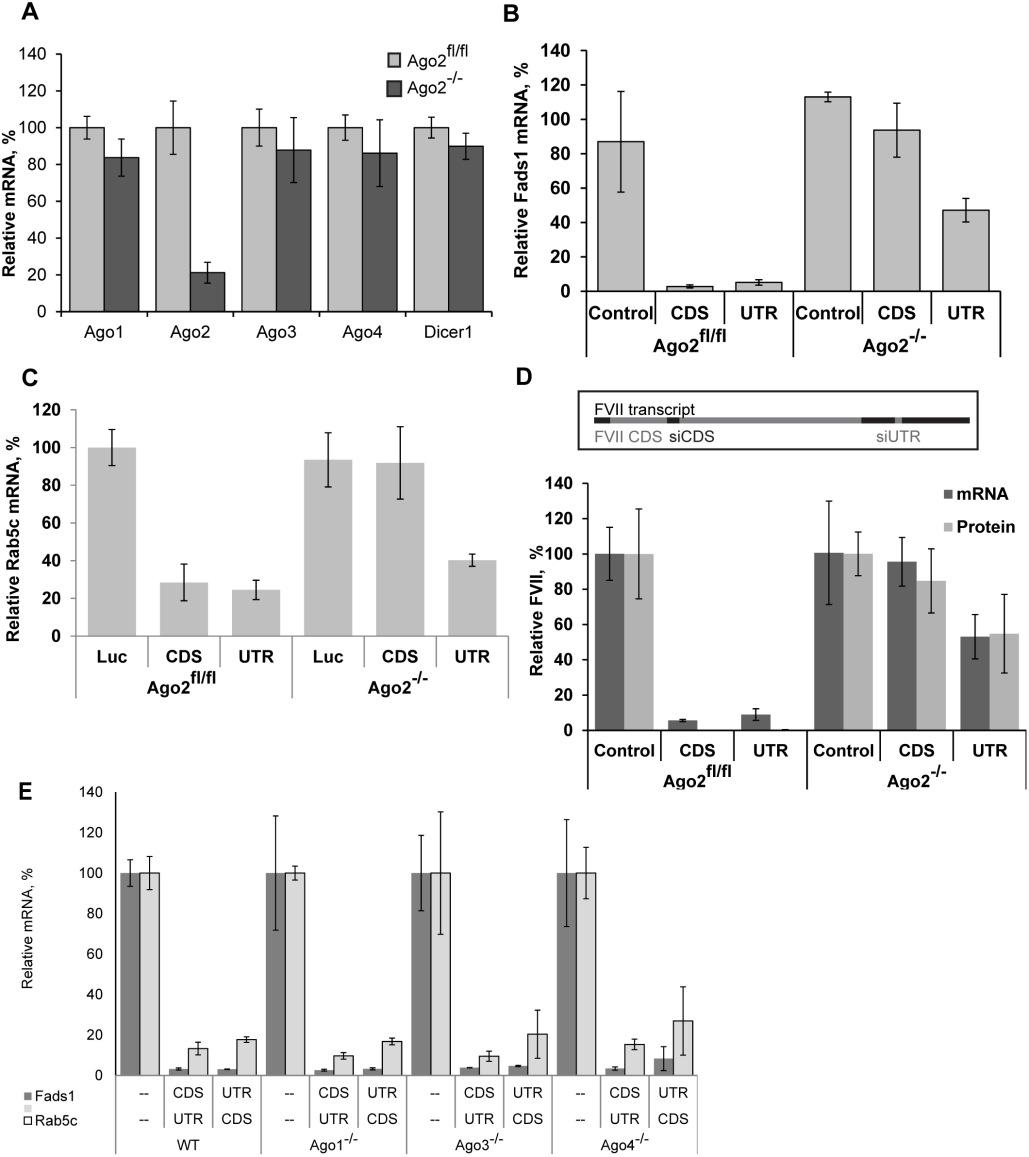
siRNAs targeting CDS and 3′UTR differ in their Ago2 dependence *in vivo*. Data points are levels of mRNA measured by qRT-PCR in the livers (harvested 24 hours post-injection) of mice i.v.-injected with LNP-formulated siRNAs, as described in Materials and Methods, expressed as a percentage of mRNA levels in control siRNA-treated animals. (A) mRNA expression of Argonautes and Dicer1 in Ago2^fl/fl^ and Ago2^−/−^ mouse liver (mean ± s.d., n = 4). (B) Levels of Fads1 mRNA in livers of Ago2^−/−^ mice and Ago2^fl/fl^ mice (mean ± s.d., n = 2–3) treated with siRNAs targeting Fads1 CDS or 3′UTR at 0.5 mg/kg. (C) Rab5c mRNA levels after treatment with siRNAs targeting its CDS or 3′UTR-far at 1 mg/kg (mean ± s.d., n = 2–3). (D) FVII mRNA in the liver of animals treated with siRNAs (mean ± s.d., n = 4–5, doses: 0.8 mg/kg for siRNA targeting CDS and 0.4 mg/kg for siRNA targeting 3′UTR) and FVII protein in the serum quantified by chromogenic assay. (E) Activity of siRNAs targeting CDS and 3′UTR in the absence of Ago1, 3, or 4. Legend in the bottom left corner of the graph also indicates the line in the X-axis describing the treatment type by gene (mean ± s.d., n = 3). Mice knockout for different individual Argonaute genes and C57BL/6 control animals were i.v.-injected with one of two combinations of LNP-formulated siRNAs targeting Fads1 and Rab5c (CDS-targeting for one gene, at 0.8 mg/kg and 3′UTR-targeting for the other gene, at 0.4 mg/kg) or with control Luciferase siRNA at 1.2 mg/kg.

Next we decided to address whether the observed difference in dependence of siRNAs targeting CDS and 3′UTR on Ago2 is reproduced *in vivo*. First, we confirmed the absence of Ago2 mRNA in liver of knockout animals (Fig. 3A). The absence of Ago2 has not led to compensatory changes in the expression of other members of the Argonaute protein family at the mRNA level (Fig. 3A). Similarly, we have seen unchanged levels of mRNA of each of the remaining Argonautes in MEF cells and mice with every single non-Slicer Ago knockout as well (Table S2). The residual expression of the mRNA of knockout genes detected by qRT-PCR in animals knockout for these genes shows the level of transcription of the truncated version of a given gene, because qPCR probes we used target exons not deleted as the result of knockout [31]. In the case of Ago2 knockout animals mRNA and protein signals may additionally originate from non-parenchymal cells in the liver, as Ago2 is hepatocyte-specific knockout.

In a recent report, Forman and colleagues have suggested that miRNA target sites in CDS and 3′UTR may differ in mechanism of action and in Argonaute proteins involved in knockdown. They have shown differences in patterns of sequence conservation between CDS and 3′UTR miRNA target sites, including absence of preference for looping and preference for greater number of bound nucleotides in CDS miRNA target sites. Together with the CDS-targeting siRNA dependence on Ago2 this led us to surmise that Ago2 may be the only Argonaute capable of targeting CDS, directed by either siRNA or miRNA. To test this hypothesis we have measured relative mRNA expression levels of Dicer1– a *let-7* CDS-target [36] – in the livers of Ago2^fl/fl^ and Ago2^−/−^ mice (Fig. 3A). No difference was observed, furthermore, the negative result of the rescue experiment with catalytically inactiveAgo2 (Figure S1) points at the Slicer activity as the prerequisite for CDS-targeted knockdown by siRNA. Thus CDS-targeted miRNAs, unlike siRNAs, do not interact exclusively with Ago2; the difference in sequence conservation patterns of CDS and 3′UTR miRNA target sites observed by Forman and colleagues may be explained by additional conservation constrains within CDS.

We compared CDS and 3′UTR siRNA target sites for the two genes – *Fads1* and *Rab5c* – in vivo by treating mice with LNP siRNA formulations (Fig. 3B, C). We show that they behave the same as they did in vitro, and that a third gene – *FVII* – has the same knockdown profile (Fig. 3D). In the *Rab5c* CDS/UTR siRNA pair the CDS duplex is less effective (IC50 values are 1 nM and 0.1 nM, respectively), so one may argue that loss of knockdown is due to insufficient dose. In contrast, both duplexes targeting *Fads1* mRNA are highly potent, so the difference in the efficacy of knockdown between duplexes targeting CDS and 3′UTR observed in this case supports the hypothesis of direct influence of position within the transcript. In addition, dose responses done in vitro (Fig. 1B, C) have demonstrated the absence of knockdown directed by the highest doses of siRNAs targeting CDS even for the most potent ones (judging by their activity in Ago2^fl/fl^ MEF cells). In fact, the distribution of duplexes in the graph on Figure 1A shows that this is true for multiple siRNAs targeting different genes: siRNAs targeting CDS form a tighter group than the ones targeting 3′UTR, all showing robust knockdown in Ago2^fl/fl^ MEF cells and insignificant knockdown in Ago2^−/−^ MEF cells. Thus, the depth of 3′UTR-targeted knockdown in the presence of Ago2 does not predict the depth of Ago2 independent mRNA knockdown.

In the absence of Ago2 CDS-targeted siRNA may influence the protein level of the targeted gene without causing its mRNA degradation, as had been shown for some miRNA, reviewed in [4]. To test the translational repression hypothesis we have measured the effects of siRNAs targeting CDS and 3′UTR on the mRNA and on the protein levels of coagulation factor VII (*FVII*) following 24 hours long knockdown. The pattern of *FVII* protein expression observed matched its mRNA expression pattern (Fig. 3D), suggesting that the main effect of siRNA in the RISC containing non-Slicer Argonautes is achieved through mRNA destabilization and degradation, rather than inhibition of translation.

For all three genes tested: *Fads1*, *Rab5c*, and *FVII* (Figure 3B, C and D, respectively) siRNAs targeting CDS had no effect on mRNA levels in Ago2^−/−^ mice, while 3′UTR-targeted knockdown in vivo was attenuated, but not abolished in the absence of Ago2. Thus, the same Ago2^−/−^ phenotype was observed *in vivo* as *in vitro*: complete loss of CDS-targeted knockdown, at the protein level, in addition to the mRNA level; partial loss of 3′UTR-targeted knockdown.

Having determined the effects of absence of Ago2 on siRNA function *in vivo*, and considering the potential role of non-Slicer Argonautes in the residual knockdown, we wanted to see if deletion of any of them will impact 3′UTR-targeted knockdown. The absence of Argonaute 1 (the most abundant of non-Slicer Argonaute proteins, see Table S2) in MEF cells had no effect on siRNA activity, irrespective of its’ targeting within the transcript (data not shown). For both genes tested *in vivo*, no difference in the efficacy of knockdown (no changes in *Fads1* or *Rab5c* mRNA recovery) with siRNAs targeting either CDS or 3′UTR was seen in the absence of Ago1, 3, or 4 (Fig. 3E). As suggested previously [14] the presence of Ago2 and its more robust Slicer-dependent activity masked the effects, if any, of non-Slicer Argonaute proteins. It has been shown previously, that overexpression of non-Slicer Argonaute proteins leads to shRNA inhibition [13], conceivably due to competition for shRNA (which they can all bind [11], [12], [16]) with the more effective Ago2 and/or due to competition for target site binding between Ago2 and non-Slicer Argonaute containing RISC complexes. Similar absence of effect of transient depletion of non-Slicer Argonautes on knockdown had been shown previously in cell culture [12], [14].

### Roles of individual non-Slicer Argonautes in siRNA-directed knockdown

As shown in Figure 1D, we have reproduced Ago2^−/−^ phenotype by siRNA-induced knockdown of Ago2 *in vitro*. Ability to do the same in animals with individual non-Slicer Argonautes’ knockouts, would allow us to test the hypothesis that one particular Argonaute protein is responsible for the observed residual activity of siRNAs targeting 3′UTR in the absence of Ago2, as has been suggested for Ago1 [14], and to determine the potential roles of non-Slicer Argonautes at physiological concentrations *in vivo*. We have selected siRNA-induced knockdown as means for depletion, as has been successfully done previously [12], [14] despite the predicted danger of undesired or unknown interference effects [37], which we were careful to minimize by utilizing the lowest effective dose, to avoid saturation of the RNAi pathway [13], [38].

#### Persistent Knockdown of Ago2 mRNA by siRNA *in vivo*

**Figure 4:**
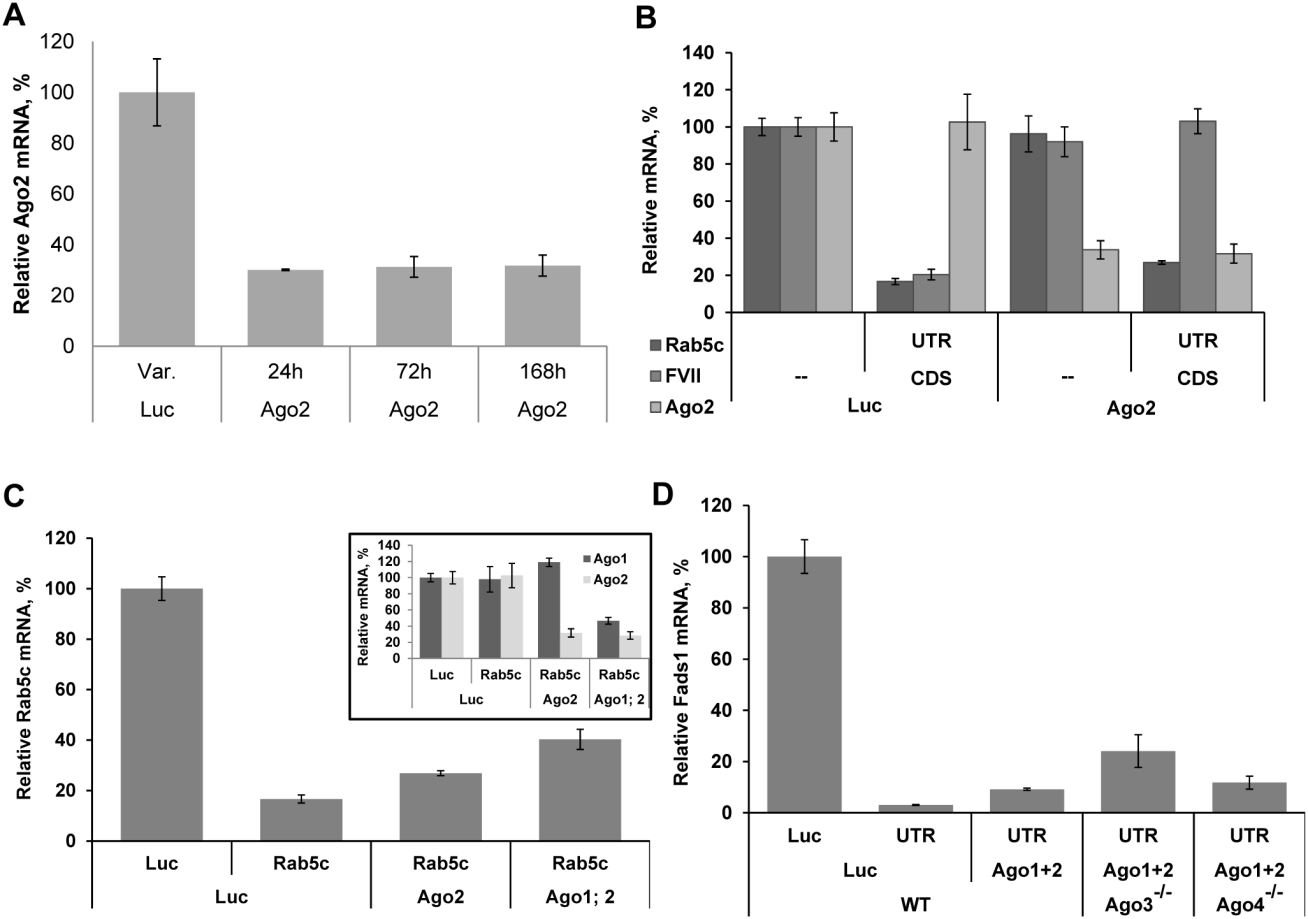
Effect of individual Argonautes in vivo. (A) C57BL/6 mice were i.v.-injected with LNP-formulated siRNA targeting Ago2–3′UTR or with control Luciferase siRNA at 0.5 mg/kg. Animals were sacrificed at indicated time points and Ago2 mRNA was quantified by bDNA assay (mean ± s.d., n = 5). (B) bDNA measurement of mRNA knockdown of Rab5c (by siRNA targeting 3′UTR-near at 0.5 mg/kg) and FVII (by siRNA targeting CDS at 0.2 mg/kg) in wild-type mice (mean ± s.d., n = 4–5) following 4 days of Ago2 knockdown (by siRNA targeting 3′UTR at 0.5 mg/kg). (C) Animals were i.v.-injected with Ago1, Ago2, or Luc siRNA on day 1, Ago1 treated animals were further i.v.-injected with Ago2 on day 2; and Rab5c-3′UTR-near siRNA was introduced on day 5 (where indicated; each siRNA was dosed at 0.5 mg/kg). All animals were sacrificed on day 6. Liver mRNA levels for Ago1, Ago2, and Rab5c were measured by bDNA assay (mean ± s.d., n = 4–5). (D) Mice were i.v.-injected with LNP containing 0.5 mg/kg siRNA against each of Ago1 and Ago2 (or 1 mg/kg of Luciferase control siRNA) twice at week long intervals, at the end of the second week mice were i.v.-injected with Fads1–3′UTR or Luciferase control siRNAs at 0.4 mg/kg. Mice were sacrificed for liver RNA extraction the day after the last injection (mean ± s.d., n = 3).

First, we confirmed ability of Ago2 siRNA to downregulate targeted mRNA level *in vivo* (Fig. 4A). Single treatment of mice with 0.5 mg/kg dose of 3′UTR-targting siRNA led to 70% reduction in Ago2 mRNA level, and practically no Ago2 mRNA restoration was observed for 7 days (Fig. 4A). Since our delivery system mainly targets hepatocytes [39], [40], non-parenchymal liver cells may contribute to the residual Ago2 signal detected in total liver samples. Persistence of knockdown may be due to the activity of non-Slicer Argonautes, rather than to the activity of a fraction of Ago2 protein remaining in hepatocytes still bound to siRNA targeting Ago2 mRNA, as the siRNA used to diminish its expression targets 3′UTR of Ago2 mRNA. We have seen comparable depth of knockdown with siRNAs targeting 3′UTRs of other genes’ mRNAs in Ago2^−/−^ MEF cells (Fig. 1A) and mice (Fig. 3C). It has been shown previously, that Ago2 protein has a relatively long half-life, on the order of days [41], [42]. Since Ago2 mRNA knockdown was persistent, in subsequent experiments we postponed the second injection (with target-gene siRNA, e.g. *Fads1*) several days in order to reach minimum protein level.

#### Ago2 knockdown reproduces Ago2^−/−^ phenotype *in vivo*

Pretreatment of wild-type C57BL/6 mice with Ago2 siRNA four days before the injection of siRNA targeting CDS of *FVII* mRNA led to complete loss of this duplex activity, while co-injected siRNA targeting 3′UTR of *Rab5c* mRNA remained active (Fig. 4B). Thus, as we have shown earlier *in vitro* (Fig. 1D), Ago2 KD with siRNA reproduces Ago2 KO phenotype *in vivo*: it leads to complete loss of CDS-targeted knockdown, and to partial loss of 3′UTR-targeted knockdown. This allows/justifies utilization of Ago2 knockdown in mice knockout for non-Slicer Argonautes, to determine their roles in the observed residual knockdown.

Since Ago1 is the most abundant of non-Slicer Argonautes, and it has previously been shown to play the major role in Ago2-independent silencing [14] we have started by measuring the efficacy of *Rab5c* 3′UTR-targeted knockdown in Ago1^−/−^ mice pre-treated with anti-Ago2 siRNA. No difference in the level of knockdown was detected between wild-type and Ago1^−/−^ mice treated with Ago2 siRNA (data not shown). We surmise that apparent absence of Ago1 role in siRNA knockdown in this case may be due to some preexisting properties, compensating for the lack of Ago1 in these animals.

One interesting potential application of Ago2 knockdown may be for confirmation that the phenotype observed upon a gene’s knockdown is not due to off-target effects [43], as they persist in its absence [14].

#### Combined Knockdown of Ago1 and Ago2 Further Decrease Rab5c mRNA Knockdown by siRNA targeting 3′UTR

Next we tested the effect of knockdown of Ago1 together with Ago2 in wild-type mice on the activity of siRNA targeting 3′UTR of *Rab5c* mRNA introduced four days later. We have previously seen no difference in knockdown between wild-type and Ago1^−/−^ mice (Fig. 3E), here, after Ago1 knockdown alone in wild-type mice, *Rab5c* knockdown result was also the same as in Luciferase siRNA pretreated mice (data not shown). Depletion of Ago1 in addition to Ago2 (unlike depletion of Ago2 in Ago1^−/−^ animals, see above) further decreased the depth of *Rab5c* knockdown (Fig. 4C), indicating Ago1 role in Slicer-independent siRNA-directed mRNA degradation. At the same time, strong residual activity of *Rab5c* 3′UTR siRNA suggested that the remaining two Argonautes are also functionally interacting with 3′UTR-targeting siRNAs. It has been reported that all miRNA species were still similarly processed and loaded onto Ago3 in Ago1/2 double knockout skin samples, suggesting that Ago3 is equally competent in the processing and loading of miRNAs as Ago1 and Ago2 and that the passenger strand cleavage activity of Ago1 and Ago2 is dispensable for the biogenesis of hundreds of miRNAs in the skin [44]. We also see Ago3 play a role in siRNA-targeted mRNA knockdown in mouse liver.

#### Ago1 and Ago3 appear to be redundant in 3′UTR-directed siRNA knockdown

To confirm the role of Ago3, combination of Ago1+2 knockdown with Ago3 or Ago4 knockout was used. The results obtained for *Fads1* mRNA knockdown by siRNA targeting 3′UTR *in vivo* support Argonautes’ redundancy, in particular the role of Ago3 in 3′UTR-targeted siRNA-directed knockdown (Fig. 4D). Minimal effect of Ago4 knockout may be due to its very low expression in liver (Table S2).

A clear trend towards *Fads1* mRNA level recovery in Ago3^−/−^ animals further supports the involvement of Ago3 in siRNA-mediated 3′UTR-targeted knockdown. Deep residual knockdown of Fads1 mRNA in the absence of three out of four Argonautes is probably due to incomplete loss of Ago1 and Ago2.

Taken together, our results lead to the following conclusions: (i) for knockdown directed by exogenous siRNA *in vivo*, miRNA-like mechanism may work in addition to Ago2 cleavage; (ii) non-Slicer Argonautes (Ago1 and Ago 3) are instrumental to the observed miRNA-like mechanism of knockdown, and (iii) they are redundant in this role in mouse liver. These conclusions provide two suggestions for future improvement of RNAi application strategy. First, it may be beneficial to target 3′UTR, rather than CDS, to turn non-Slicer Argonautes from siRNA deposit/sequestration platforms [13] into active participants of knockdown [14], [24]. Second, it may be useful to take into account the stability and persistence of loaded RISC complexes [8], [42], [45], in particular in quiescent cells [46], when frequency and dosage of siRNA treatment are selected [47]–[49].

## Materials and Methods

### siRNA design, synthesis, selection, and formulation

We selected the candidate 21-mer siRNAs with maximum target transcript specificity as described [49]. Mouse mRNA sequences (listed in Table S1) were used to select appropriate candidate target sequences for their corresponding siRNAs. Single-stranded chemically modified RNAs were synthesized at Alnylam Pharmaceuticals (Cambridge, MA) using standard phosphoramidite chemistry. Deprotection and purification of the crude oligoribonucleotides by anion exchange HPLC were carried out according to established procedures. siRNA duplexes were generated by annealing equimolar amounts of complementary sense and antisense strands. All siRNAs used in this study are listed in Table S1.

*FVII*-CDS- and luciferase-specific control siRNAs were described earlier [50]. siRNAs specific to murine *Ago1*, *Ago2*, *FVII*-3′UTR, *Fads1*, and *Rab5c* mRNAs were identified by screening sets of 20–30 siRNA duplexes per gene. Best duplexes were selected by transfection of NIH/3T3 cells and IC50 values determined. siRNA specific to *FVII*-3′UTR were identified similarly, except cultured primary mouse hepatocytes were used for screening. Duplexes selected for each target were synthesized on a larger scale at Alnylam Pharmaceuticals (Cambridge, MA) and were characterized by electrospray mass spectrometry and anion exchange HPLC prior to their use in LNP preparation, which was performed as described [40], [51].

### In vitro studies

Derivation and manipulation of mouse embryonic fibroblast (MEF) cell lines with individual Argonaute genes’ deletions were described previously [31]. MEF cells were maintained in Dulbecco’s modified Eagle’s medium (Life Technologies) supplemented with Non-Essential Amino Acids, Penicillin, Streptomycin, and 15% fetal bovine serum. For transfection Lipofectamine RNAiMAX (Life Technologies) was used following manufacturer’s protocol for reverse transfection, MEF cells were plated at average density of 100000 cells/cm^2^; siRNA concentrations are indicated in the text. Target genes’ mRNA knockdown following transfections was analyzed by branched DNA assay as described [52] or by quantitative reverse transcription real-time PCR (qRT-PCR) measurement of target genes’ mRNA levels using TaqMan gene-specific probes (Applied Biosystems) on a LightCycler 480 II (Roche). GAPDH mRNA levels were used for data normalization. The probes used in the bDNA assay were QuantiGene 2.0 RNA Probe Sets for mouse *FVII*, *Fads1*, *Fads2*, *Rab5a*, *Rab5c*, and *Gapdh* mRNAs (Affymetrix).

For rescue experiment pMigR vectors bearing wild-type or D669A mouse Ago2 coding sequences [31] were transfected with Fugene HD into Ago2^−/−^ MEF cells, followed by reverse transfection with siRNA the next day.

*Rab5c* 5′RACE was done with GeneRacer kit (Life Technologies), essentially as described previously [49], on 2 µg of the total RNA isolated from Ago2^fl/fl^ MEF cells and Ago2^−/−^ MEF cells transfected with 10 nM *Rab5c* 3′UTR-near or Luciferase control siRNA, avoiding dephosphorylation and decapping steps. Gene specific primers used for 5′RACE were: Rab5c-GSPrev-1198 (for first round of PCR) 5′-GCAAGAAGGGAAGAAAGGGTGACT-3′ and Rab5c-GSPrev-1020 (for Nested PCR) 5′-ACAGAAAGGTGCAGGTGGAATAACTC-3′. “A Plasmid Editor” program by M. Wayne Davis (http://biologylabs.utah.edu/jorgensen/wayned/ape/) was used for primer design and sequence alignment.

### Animal studies

All experiments done in wild-type animals were conducted at Alnylam Pharmaceuticals and strictly followed institutional, federal, state and local guidelines and were approved by Institutional Animal Care and Use Committee (AAALAC Unit Number 001345, NIH assurance number A4517-01). All the animals were kept in a conventional barrier animal facility with a climate-controlled environment having 12-hour light/dark cycles in polystyrene cages containing wood shavings, fed standard rodent chow and water.

For the experiments, female 8 weeks old C57BL/6 mice were purchased from Charles River Laboratories. For silencing experiments mice were i.v. bolus injected (10 µL/g; maximum total amount of siRNA delivered per injection was 1.2 mg/kg and in each experiment all animals received the same total dose) and sacrificed by CO_2_ overdose before tissue harvest. Chromogenic assay for quantification of Factor VII protein in mouse serum and bDNA assay for measurement of mRNA in mouse liver have been done as recently described [52]. The probes used in the bDNA assay are listed above.

All experiments done in knockout animals were conducted at Rockefeller University. Mice were housed under specific pathogen-free conditions and experimental protocols were approved by the Rockefeller University Institutional Animal Care and Use Committee. Individual Argonautes’ floxed models were described previously [31]. In the case of Ago2 the mice with a floxed gene were bred to Alb-Cre (obtained from Jackson Labs) for the hepatocyte conditional deletion. All mice were bred onto a C57BL/6 background. *In vivo* experiments in knockout animals were carried out in a blinded fashion. Total RNA was isolated from snap-frozen, ground liver samples with RNeasy 96 Universal Tissue kit (Qiagen) as described by the manufacturer, reverse transcribed with High Capacity cDNA Reverse Transcription Kit (Applied Biosystems) and used for qRT-PCR measurement of target genes’ mRNA levels using TaqMan gene-specific probes (Applied Biosystems) on a LightCycler 480 II (Roche). GAPDH mRNA levels were used for data normalization.

### Western blot analysis

For western blot analysis whole liver lysates were prepared from liver powders from control, siRNA-treated, and knockout animals, 30 µg of total protein (measured by BCA assay) run on SDS-PAGE, transferred to nitrocellulose membranes and incubated with antibodies to Ago2 RN029PW (MBL Ribonomics). beta-Actin levels (clone AC-15, Sigma-Aldrich cat.no.A1978) were used to normalize for protein loading. Protein bands were visualized by using LI-COR Odyssey infrared imaging system (with appropriate secondary antibodies: goat anti-rabbit IgG IRDye 800CW and goat anti-mouse IgG IRDye 680LT).

## Supporting Information

Figure S1 **Ago2 rescue experiment.** MEF Ago2^−/−^ cells were transiently transfected with Ago2 wild-type or Ago2(D669A) Slicer-incompetent mutant, followed after 24 hours by a transfection with serial dilutions of Fads1-CDS or Fads1-3′UTR targeting siRNAs. (A) Fads1 (and Ago2) mRNA levels were determined by qRT-PCR. Three independent transfections were done in duplicate; averages of duplicates of each transfection are shown. Western blot (B) and qRT-PCR (C) analyses of wild-type and D669A mutant Ago2 expression levels.
(PPT)

Figure S2 **Features of siRNA-mediated degradation of mRNA in the absence of Ago2.** (A) Deeper knockdown is achieved by 24 h compared to 3 h in all Rab5c siRNA treated cells, except MEF Ago2^−/−^ treated with CDS-targeting siRNA, where no significant knockdown is detected at either time point. Data is presented as mean ± s.d. for two technical replicates of bDNA measurement. (B) Complete image of the 5′RACE Nested PCR electrophoresis, shown in FIGURE 2B, includes two different control reactions for each cell type.
(PPT)

Figure S3 **5′RACE products’ sequences alignment.** Sequenced cloned PCR products were aligned to mouse Rab5c mRNA NM_024456.3 (by APE software). mRNA sequence complementary to Rab5c-3′UTR-near siRNA is highlighted green (cleavage site marked with an asterisk).
(PPT)

Table S1 **Sequences and relative target-site positions of siRNAs.**
(XLS)

Table S2 **Relative Argonaute mRNA abundance.**
(XLS)
