# Supplementary material for: The Roles of Individual Mammalian Argonautes in RNA Interference In Vivo
Source: PLoS One. 2014 Jul 3;9(7):e101749. doi: 10.1371/journal.pone.0101749 (PMC4081796; doi:10.1371/journal.pone.0101749)
Supplement: Figure S1 — Ago2 rescue experiment. MEF Ago2−/− cells were transiently transfected with Ago2 wild-type or Ago2(D669A) Slicer-incompetent mutant, followed after 24 hours by a transfection with serial dilutions of Fads1-CDS or Fads1-3′UTR targeting siRNAs. (A) Fads1 (and Ago2) mRNA levels were determined by qRT-PCR. Three independent transfections were done in duplicate; averages of duplicates of each transfection are shown. Western blot (B) and qRT-PCR (C) analyses of wild-type and D669A mutant Ago2 expression levels. (PPT) [file pone.0101749.s001.ppt]

## Slide 1
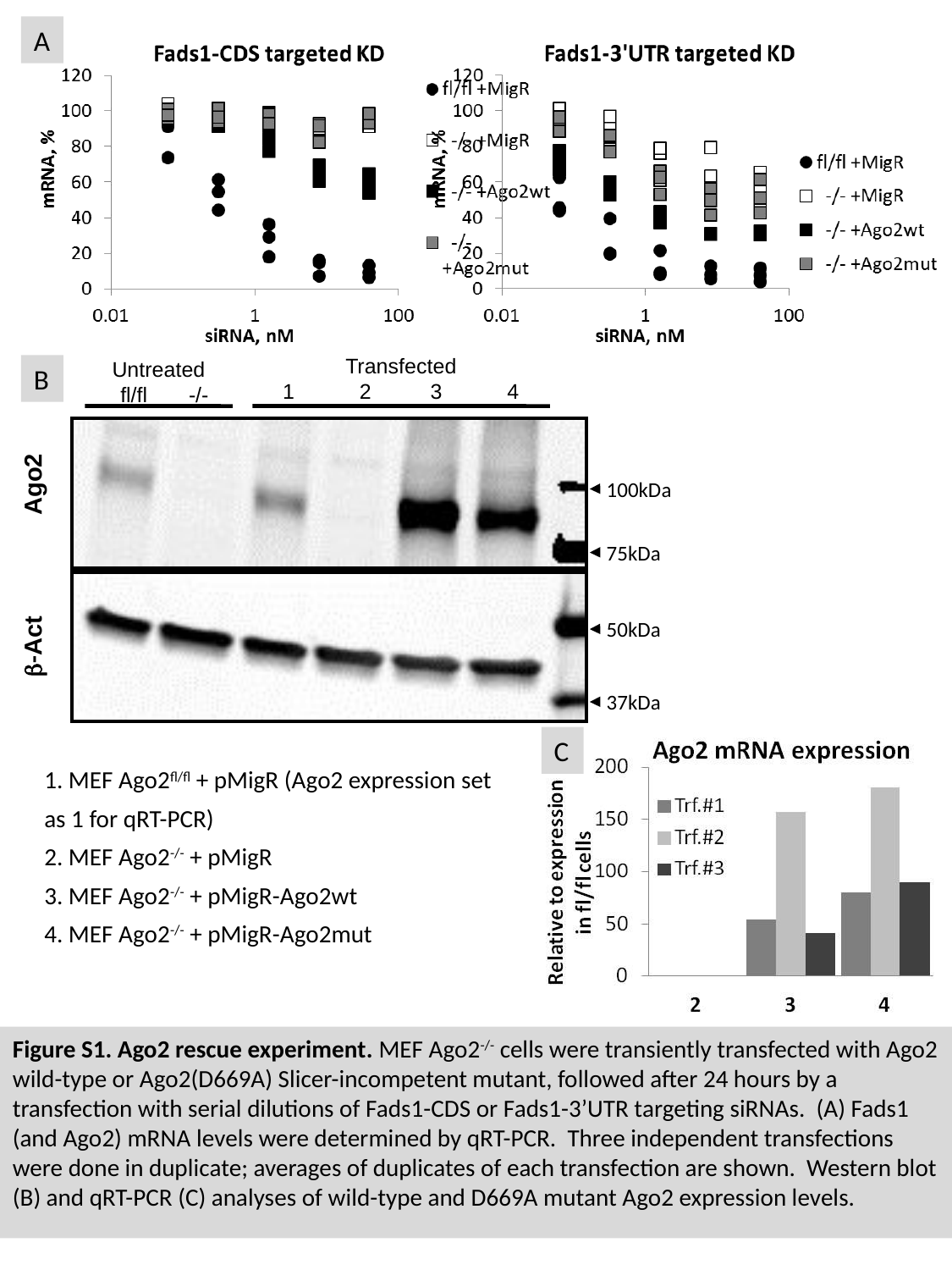

A
Transfected
1 2 3 4
Untreated
 fl/fl -/-
Ago2
100kDa
75kDa
50kDa
β-Act
37kDa
B
C
1. MEF Ago2fl/fl + pMigR (Ago2 expression set as 1 for qRT-PCR)
2. MEF Ago2-/- + pMigR
3. MEF Ago2-/- + pMigR-Ago2wt
4. MEF Ago2-/- + pMigR-Ago2mut
Figure S1. Ago2 rescue experiment. MEF Ago2-/- cells were transiently transfected with Ago2 wild-type or Ago2(D669A) Slicer-incompetent mutant, followed after 24 hours by a transfection with serial dilutions of Fads1-CDS or Fads1-3’UTR targeting siRNAs. (A) Fads1 (and Ago2) mRNA levels were determined by qRT-PCR. Three independent transfections were done in duplicate; averages of duplicates of each transfection are shown. Western blot (B) and qRT-PCR (C) analyses of wild-type and D669A mutant Ago2 expression levels.
