# Supplementary material for: The Roles of Individual Mammalian Argonautes in RNA Interference In Vivo
Source: PLoS One. 2014 Jul 3;9(7):e101749. doi: 10.1371/journal.pone.0101749 (PMC4081796; doi:10.1371/journal.pone.0101749)
Supplement: Figure S2 — Features of siRNA-mediated degradation of mRNA in the absence of Ago2. (A) Deeper knockdown is achieved by 24 h compared to 3 h in all Rab5c siRNA treated cells, except MEF Ago2−/− treated with CDS-targeting siRNA, where no significant knockdown is detected at either time point. Data is presented as mean ± s.d. for two technical replicates of bDNA measurement. (B) Complete image of the 5′RACE Nested PCR electrophoresis, shown in FIGURE 2B, includes two different control reactions for each cell type. (PPT) [file pone.0101749.s002.ppt]

## Slide 1
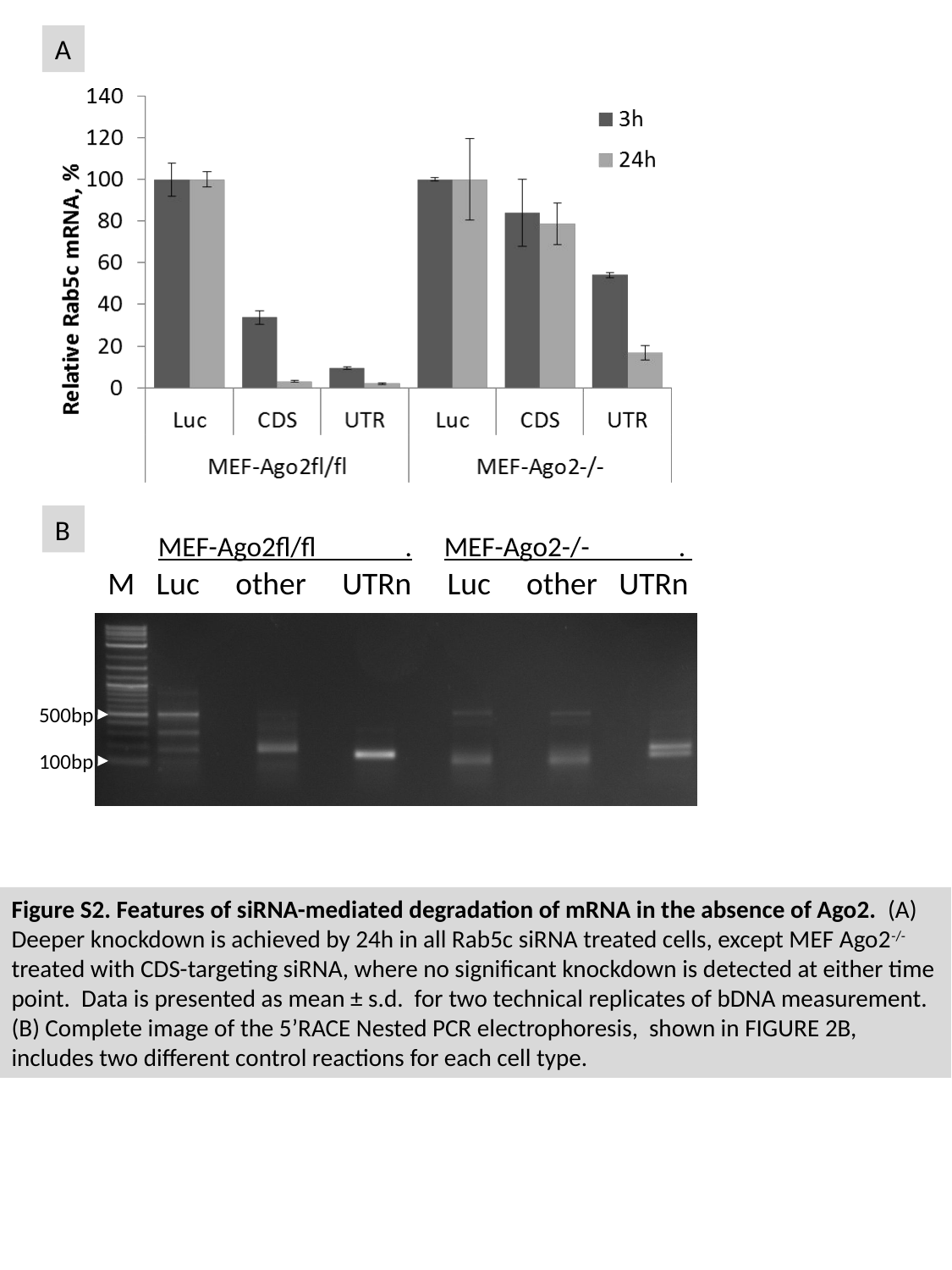

A
B
 MEF-Ago2fl/fl . MEF-Ago2-/- .
M Luc other UTRn Luc other UTRn
500bp
100bp
Figure S2. Features of siRNA-mediated degradation of mRNA in the absence of Ago2. (A) Deeper knockdown is achieved by 24h in all Rab5c siRNA treated cells, except MEF Ago2-/- treated with CDS-targeting siRNA, where no significant knockdown is detected at either time point. Data is presented as mean ± s.d. for two technical replicates of bDNA measurement. (B) Complete image of the 5’RACE Nested PCR electrophoresis, shown in FIGURE 2B, includes two different control reactions for each cell type.
