# Supplementary material for: The Roles of Individual Mammalian Argonautes in RNA Interference In Vivo
Source: PLoS One. 2014 Jul 3;9(7):e101749. doi: 10.1371/journal.pone.0101749 (PMC4081796; doi:10.1371/journal.pone.0101749)
Supplement: Figure S3 — 5′RACE products’ sequences alignment. Sequenced cloned PCR products were aligned to mouse Rab5c mRNA NM_024456.3 (by APE software). mRNA sequence complementary to Rab5c-3′UTR-near siRNA is highlighted green (cleavage site marked with an asterisk). (PPT) [file pone.0101749.s003.ppt]

## Slide 1
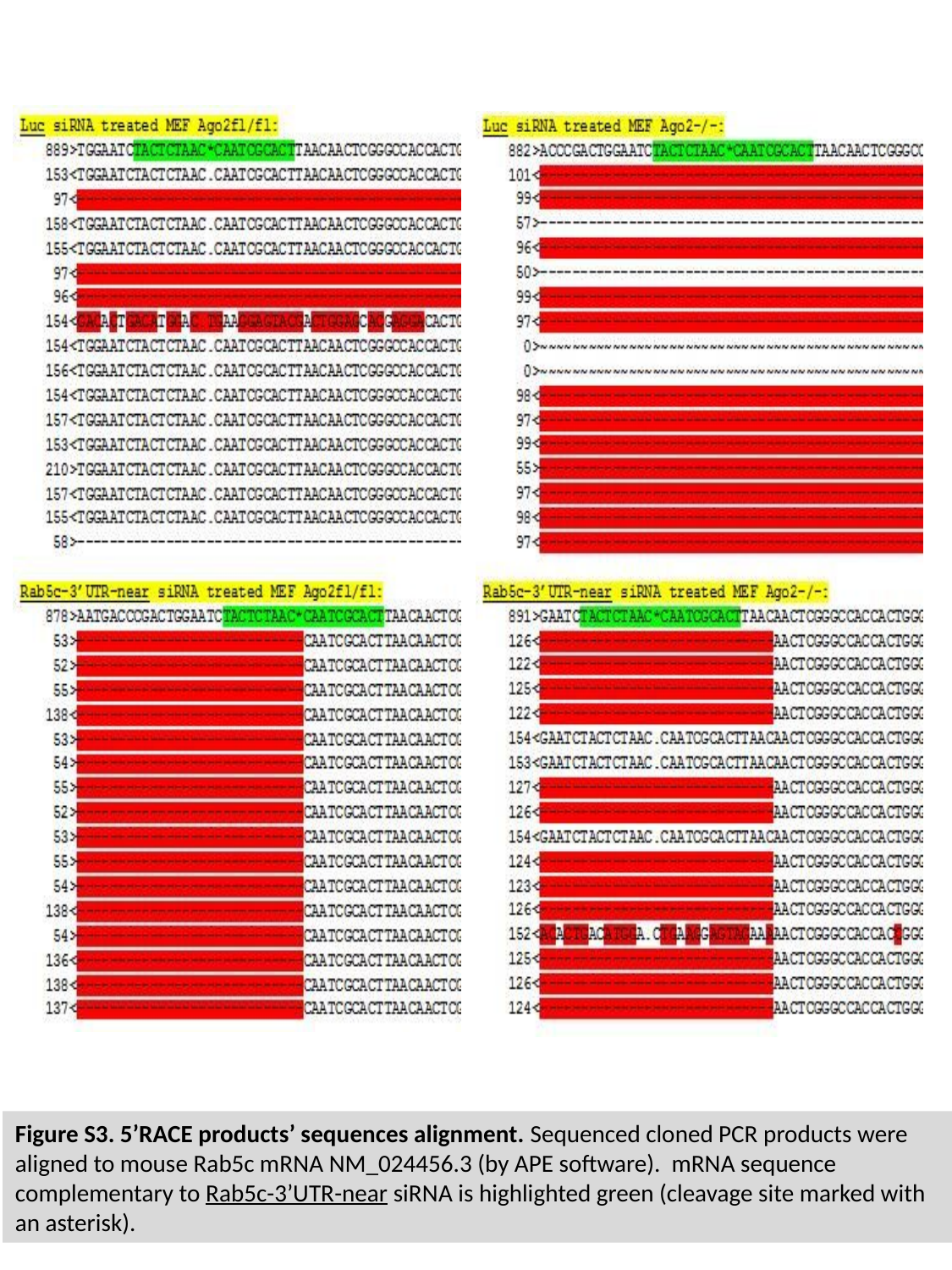

Figure S3. 5’RACE products’ sequences alignment. Sequenced cloned PCR products were aligned to mouse Rab5c mRNA NM_024456.3 (by APE software). mRNA sequence complementary to Rab5c-3’UTR-near siRNA is highlighted green (cleavage site marked with an asterisk).
